# Extremophilic Microalgae and Cyanobacteria as Platforms for Climate Resilience, Circular Resource Utilization and Bioprospecting

**DOI:** 10.3390/microorganisms14092113

**Published:** 2026-09-21

**Authors:** Mixue Wang, Yan Wang, Die Zhou, Yu Xie, Shoukai Guo, Zhongliang Sun

**Affiliations:** 1 School of Biomedicine and Pharmacy, Jiangsu Agri-Animal Husbandry Vocational College, Taizhou 225300, China; 2016020321@jsahvc.edu.cn (M.W.);; 2 College of Life Sciences, Yantai University, Yantai 264005, China

**Keywords:** carbon capture, circular resource utilization, bioprospecting, ecological niche, extreme environment

## Abstract

The untapped genetic diversity of extremophilic microalgae provides distinctive physiological and biochemical traits with potential to support Sustainable Development Goal (SDG) 6 (Clean Water and Sanitation), SDG 7 (Affordable and Clean Energy), and SDG 15 (Life on Land). This review examines the diversity, stress-adaptation mechanisms, and biotechnological potential of extremophilic microalgae, with emphasis on their roles in resource recovery, carbon utilization, and bioproduct generation. Their ability to maintain cellular functions under extreme salinity, pH, temperature, radiation, and nutrient conditions enables their cultivation in environments that may be unsuitable for conventional production systems and facilitates the accumulation of lipids, pigments, carbohydrates, proteins, and other bioactive metabolites. These characteristics create opportunities to couple wastewater treatment, carbon capture and utilization, and biomass valorization within circular bioprocesses. However, laboratory-scale demonstrations should not be equated with established environmental or commercial benefits, as strain-specific physiological requirements, energy demand, process control, harvesting, downstream processing, and resource inputs can constrain scale-up. The review therefore emphasizes the need to evaluate extremophilic microalgal systems using integrated techno-economic and life-cycle approaches rather than biological productivity alone. Future advances will depend on combining multi-omics, metabolic engineering, adaptive cultivation, process optimization, and predictive modeling to identify robust strains and cultivation strategies. Such integration could accelerate the translation of extremophile-based bioprocesses into resource-efficient and economically credible applications for environmental sustainability.

## 1. Introduction

The continuous growth in the world population is exerting pressure on resources, thus creating competition for essential commodities such as food, water, and energy [1]. This competition has contributed to environmental degradation and loss of biodiversity through unsustainable practices and overexploitation of natural resources to meet increasing demand [2]. To counteract the negative effects associated with resource overexploitation, there is a need for sustainable practices and conservation strategies that ensure the long-term availability of these resources for future generations [3]. Different strategies, including the promotion of renewable energy sources, resource-efficient agricultural practices, and protection of natural habitats, have been implemented to mitigate the environmental consequences of resource competition [4]. However, pressure on land and water resources continues because of increasing demand for food, energy, and industrial production [5]. Climate change further intensifies these pressures by altering temperature and precipitation regimes, increasing water scarcity, and accelerating ecosystem degradation. These challenges have prompted a shift towards circular economy models and ecosystem-based solutions that seek to decouple economic development from the continued consumption of finite resources. Such approaches require biological production platforms capable of recovering resources from waste streams, functioning under non-conventional conditions, and generating products with environmental and economic value [6]. The bioprospecting potential of microalgae has been explored as a biological alternative for reducing pressure on conventional resource-intensive production systems [7]. This is attributed to their high biomass productivity, photosynthetic carbon fixation, and ability to accumulate diverse primary and secondary metabolites, increasing their value for pharmaceutical, nutraceutical, food, cosmetic, and bioenergy applications [8]. The ability of some microalgae to grow on non-arable land and utilize saline, brackish, or wastewater streams may reduce competition with conventional agriculture for freshwater and fertile land [9]. Microalgal cultivation can therefore provide opportunities to couple biomass production with wastewater treatment, nutrient recovery, carbon utilization, and bioproduct generation. However, these advantages should not be considered universal, because the environmental and economic performance of microalgal systems depends strongly on species, cultivation conditions, downstream processing, and the characteristics of the resource stream being used. Moreover, environmental conditions such as high temperature, low nutrient availability, extreme pH, and high salinity can substantially affect microalgal growth, photosynthesis, and metabolic activity [10]. These conditions may alter physiological and metabolic processes and, depending on their intensity and duration, reduce biomass productivity or redirect carbon flux towards stress responses rather than target-product formation [11]. Consequently, the development of resilient cultivation systems has become an important research priority, with genetic engineering, metabolic engineering, adaptive laboratory evolution, and process optimization being investigated to improve stress tolerance and product formation [12].

Despite these advances, much of the microalgal biotechnology literature has focused on comparatively well-characterized mesophilic taxa and controlled cultivation environments. Extremophilic microalgae, by contrast, have evolved under persistent or recurrent environmental stresses and occupy ecological niches that include hypersaline lakes, hot springs, acidic and alkaline waters, high-irradiance environments, and highly contaminated aquatic systems [13]. Their habitat flexibility and physiological plasticity enable selected taxa to maintain cellular functions under conditions that can inhibit many conventional microorganisms. These characteristics provide a distinct biological resource for bioprospecting because environmental stress can act not only as a constraint but also as a selective pressure that induces specialized physiological responses and metabolite production. For example, extremophilic microalgae may accumulate compatible solutes, pigments, lipids, carbohydrates, extracellular polymeric substances, and other stress-associated compounds that contribute to cellular protection and may possess biotechnological value. However, the presence of a stress-responsive metabolite does not necessarily translate into high productivity, commercial feasibility, or environmental superiority; these outcomes depend on cultivation intensity, biomass yield, product recovery, and downstream processing requirements [14]. The ability of extremophilic microalgae to tolerate specific combinations of high salinity, temperature, acidity, alkalinity, irradiance, or pollutant exposure also creates opportunities for cultivation in environments that are unsuitable for many conventional production organisms [15]. For example, selected extremophilic taxa can be cultivated using saline water, acidic process streams, industrial effluents, or other unconventional resources, potentially reducing dependence on freshwater and arable land. Their tolerance to physicochemical conditions that suppress competing microorganisms may also facilitate cultivation under less stringent contamination-control requirements; however, this does not eliminate contamination risks or guarantee lower operating costs, particularly when temperature, pH, light, gas transfer, or nutrient conditions require active control [16]. Extremophilic microalgae are therefore better viewed as specialized biological platforms whose value derives from their capacity to operate within particular environmental windows rather than as universally superior alternatives to conventional microalgae. This distinction is important when assessing their contribution to circular bioeconomy systems, because the sustainability of an extremophilic process ultimately depends on the balance between resource inputs, environmental performance, product yields, and process scale [17].

Beyond their cultivation characteristics, extremophilic microalgae possess diverse metabolic repertoires shaped by long-term adaptation to environmental stress. Their responses to extreme conditions can involve changes in membrane composition, antioxidant systems, osmotic regulation, photosynthetic machinery, carbon metabolism, and secondary metabolism. These responses can generate or enrich molecules with potential applications as pigments, lipids and fatty acids, carbohydrates and polysaccharides, compatible solutes, antioxidants, enzymes, and other bioactive compounds [18]. Their metabolic diversity therefore provides an important basis for bioprospecting, particularly where conventional organisms do not readily produce the required compound or cannot maintain production under the relevant process conditions. At the same time, the extent to which stress-induced metabolite accumulation can be reproduced, optimized, and maintained at industrial scale remains an important knowledge gap [19].

The potential relevance of extremophilic microalgae extends beyond individual products to integrated resource-utilization systems. Their cultivation can potentially couple carbon fixation with nutrient recovery and biomass production, while the resulting biomass may serve as a feedstock for sequential extraction of high-value compounds and subsequent conversion of residual fractions into fuels, materials, or other products. Such integration is consistent with circular-bioeconomy principles because it seeks to maximize resource recovery and minimize residual waste rather than relying on a single product pathway [20]. Extremophilic systems may also provide opportunities for utilizing saline or otherwise unsuitable water resources and for converting selected industrial or agricultural waste streams into biological products. Nevertheless, the environmental benefits of these approaches must be demonstrated through appropriate mass balances, life-cycle assessment, techno-economic analysis, and comparison with conventional cultivation systems rather than inferred solely from laboratory-scale removal or productivity values [21]. The relevance of these characteristics is particularly important under climate change, where production systems are increasingly exposed to temperature extremes, water scarcity, salinity, and other environmental disturbances. Extremophilic microalgae may provide useful biological traits for developing cultivation platforms capable of maintaining selected functions under such stresses, while their metabolites may contribute to applications in energy, agriculture, environmental remediation, and high-value biomanufacturing [16]. Their potential contribution to the Sustainable Development Goals (SDGs), particularly those related to clean water and sanitation (SDG 6), affordable and clean energy (SDG 7), and life on land (SDG 15), should therefore be interpreted as an application-level opportunity rather than an intrinsic property of extremophilic microalgae. Achieving these outcomes will depend on whether their biological advantages can be translated into processes that are technically feasible, economically competitive, and environmentally sustainable [22].

Despite growing interest, important gaps remain in connecting the ecological adaptations of extremophilic microalgae with their practical bioprospecting and industrial potential. Evidence is dispersed across studies examining individual species, stress responses, metabolites, cultivation systems, and environmental applications, making it difficult to identify which adaptations provide demonstrated technological advantages and which remain largely conceptual. In addition, trade-offs between stress tolerance, biomass productivity, metabolite accumulation, energy demand, downstream recovery, and long-term process stability are rarely considered within a unified framework. Addressing these gaps is essential for determining whether extremophilic microalgae can progress from promising biological resources to scalable platforms for climate-resilient and circular biotechnology [23]. Accordingly, this review examines the bioprospecting potential of extremophilic microalgae and cyanobacteria from the perspectives of climate resilience, circular resource utilization, carbon management, and high-value bioproduct generation. It synthesizes current evidence on their ecological significance, resource-recovery capabilities, carbon capture and utilization potential, and production of lipids, carbohydrates, pigments, and other bioactive compounds, while distinguishing experimentally demonstrated applications from emerging opportunities. The review further evaluates the biological, technological, economic, and environmental limitations affecting scale-up and identifies research priorities involving multi-omics, metabolic and adaptive engineering, process intensification, predictive modeling, life-cycle assessment, and techno-economic analysis. By linking extremophile adaptation with circularity and climate resilience, this review aims to clarify where these organisms offer genuine advantages, where evidence remains insufficient, and how their biological diversity can be translated into robust and sustainable biotechnological systems.

## 2. Methodology

A comprehensive literature assessment was conducted to synthesize current knowledge on the ecological, physiological, biochemical, and biotechnological significance of extremophilic microalgae and cyanobacteria, with particular emphasis on their potential for climate resilience, circular resource utilization, carbon capture and utilization, and bioprospecting of high-value products. The literature search primarily covered publications from 2010 to 2026 to capture recent advances in extremophile biotechnology, while earlier seminal studies were retained where they provided foundational evidence on the physiology, adaptation mechanisms, cultivation, or bioproduct potential of relevant organisms. Peer-reviewed articles were retrieved primarily from the Scopus and Web of Science databases, supplemented, where necessary, by relevant book chapters and other authoritative scholarly sources not indexed in these databases. Studies were selected based on their relevance to extremophilic microalgae and cyanobacteria, including their adaptation to extreme environments; resource recovery from wastewater and other unconventional streams; carbon fixation and utilization; production of lipids, carbohydrates, pigments, extracellular polymeric substances, and other bioactive compounds; and applications in bioenergy, bioremediation, agriculture, pharmaceuticals, and circular bioeconomy systems. Particular attention was given to studies providing experimental evidence on physiological responses, metabolite accumulation, cultivation performance, environmental applications, and product yields. Studies based solely on general microalgal systems were used selectively where they provided essential mechanistic or comparative context. The literature search was performed using combinations of English-language keywords, including extremophilic microalgae, extremophilic cyanobacteria, extremophile biotechnology, extreme environments, halophilic microalgae, thermophilic microalgae, acidophilic microalgae, alkaliphilic microalgae, microalgal bioprospecting, stress adaptation, stress-induced metabolites, circular bioeconomy, resource recovery, wastewater treatment, carbon capture, carbon utilization, carbon sequestration, CO_2_ fixation, lipids, fatty acids, carbohydrates, exopolysaccharides, pigments, bioactive compounds, biofuels, “biomaterials, bioremediation, nutraceuticals, pharmaceuticals, techno-economic analysis, and life-cycle assessment. The retrieved literature was critically evaluated to identify established applications, emerging opportunities, knowledge gaps, and technological limitations, with emphasis on distinguishing experimentally demonstrated benefits from proposed or prospective applications.

## 3. Diversity and Adaptation Mechanisms of Extremophilic Microalgae and Cyanobacteria

The ability of extremophilic microalgae to thrive under environmental conditions that are physiologically restrictive for most organisms provides an important basis for their ecological distribution and biotechnological interest [24]. They occur across diverse extreme habitats, including hypersaline lakes, hot springs, polar regions, acidic or alkaline waters, and other environments characterized by combinations of temperature, pH, salinity, irradiance, desiccation or metal stress [25]. Importantly, extremophily should be distinguished from extremotolerance and transient physiological acclimation. In this review, an extremophile refers to an organism adapted to grow and function optimally under an extreme environmental condition, whereas an extremotolerant organism can withstand an extreme condition but generally has its optimum outside that range. By contrast, a conventional biotechnology strain exposed experimentally to salinity, nutrient limitation, radiation or another stress is considered a stress-responsive organism rather than an extremophile unless its natural ecological association and sustained growth under the corresponding extreme condition have been demonstrated [15]. This distinction is important because stress-induced accumulation of a metabolite does not, by itself, establish evolutionary adaptation to an extreme habitat. The organisms considered in this review include both eukaryotic microalgae and prokaryotic cyanobacteria. These groups are therefore distinguished taxonomically throughout the text, although they are considered together where their ecological functions or adaptive strategies overlap. Cyanobacteria such as *Synechococcus* sp., *Mastigocladus* sp., and *Arthrospira* sp. are photosynthetic prokaryotes and should not be classified as microalgae in the strict taxonomic sense [26]. The collective term ‘extremophilic photosynthetic microorganisms’ is therefore used where both groups are discussed. Their diversity reflects adaptation to both individual and combined environmental extremes, with polyextremophiles representing organisms capable of maintaining growth under combinations of stresses such as high temperature and low pH or high salinity and high irradiance [27]. Thus, rather than representing discrete and mutually exclusive categories, extreme habitats form overlapping environmental gradients that select for distinct combinations of molecular, cellular and physiological traits. Thermophilic and thermoacidophilic microalgae, for example, are associated with geothermal environments, whereas psychrophilic and psychrotolerant taxa occur in polar and high-altitude habitats. Acidophilic and alkaliphilic organisms occupy environments characterized by low and high pH, respectively, while halophilic and halotolerant taxa occur in hypersaline systems. Among these groups, members of the Cyanidiales provide well-characterized examples of polyextremophily, because taxa such as *Cyanidium caldarium*, *Galdieria sulphuraria* and *Cyanidioschyzon merolae* occur in hot, acidic environments and can tolerate additional stresses, including elevated metal concentrations [27,28].

### 3.1. Thermophilic and Thermoacidophilic Microalgae and Cyanobacteria

Thermophilic microalgae can thrive at elevated temperatures, particularly in geothermal and hot-spring environments. *Cyanidium caldarium* and *Galdieria sulphuraria* are representative thermoacidophilic red microalgae associated with acidic geothermal habitats. Their classification is supported by their natural occurrence and sustained growth under combinations of high temperature and low pH rather than by short-term exposure experiments. *C. caldarium*, for example, has been characterized as both thermophilic and strongly acidophilic, with favorable growth reported around 45–50 °C and pH 3 [28]. *G. sulphuraria* similarly occurs in acidic geothermal environments and exhibits substantial metabolic flexibility, including photoautotrophic, heterotrophic and mixotrophic growth [29]. Thermophilic cyanobacteria should be considered separately from eukaryotic microalgae because species belonging to genera such as *Synechococcus* and *Mastigocladus* are photosynthetic prokaryotes that can inhabit high-temperature environments and possess cellular and biochemical adaptations that support growth at elevated temperatures. Their inclusion in this review therefore reflects their ecological and functional relevance to extremophilic photosynthetic biotechnology rather than taxonomic classification as microalgae [26]. Adaptation to high temperature involves multiple interacting mechanisms rather than a single heat-resistance trait. These include stabilization of proteins and photosynthetic complexes, regulation of membrane properties, antioxidant protection, maintenance of intracellular pH and ion homeostasis, and adjustment of central metabolism. Importantly, the presence of a thermostable enzyme alone should not be interpreted as evidence that an organism is thermophilic because the organism’s ecological origin, growth optimum and sustained physiological performance are important indicators to be considered. For example, biochemical studies of *C. caldarium* have demonstrated enhanced thermal stability of ribulose-1,5-bisphosphate carboxylase relative to enzymes from mesophilic *Chlorella*, illustrating how protein-level properties can contribute to functioning at elevated temperatures [30].

### 3.2. Psychrophilic and Cold-Adapted Microalgae

Polar and high-altitude ecosystems harbor microalgae adapted to low temperatures, including taxa associated with sea ice and snow environments. *Chlamydomonas nivalis* and *Phaeocystis antarctica* are examples of cold-adapted microalgae occurring in polar environments [31], while *Fragilariopsis cylindrus* is a well-characterized sea-ice diatom [32]. However, cold adaptation should be interpreted in relation to an organism’s natural habitat and growth characteristics rather than simply its ability to survive exposure to temperatures below 5 °C. Cold adaptation involves membrane lipid remodeling, production of ice-binding or antifreeze proteins, regulation of photosynthesis, modification of metabolic rates and enhanced control of oxidative stress [33]. In *F. cylindrus*, antifreeze proteins provide a particularly clear molecular example of adaptation to sea-ice conditions. Uhlig et al. [32] reported two antifreeze protein isoforms with thermal hysteresis values of up to 1.53 ± 0.53 °C and 2.34 ± 0.25 °C, respectively, together with distinct effects on ice-crystal morphology. These findings provide direct functional evidence that ice-binding proteins can modify ice growth and may contribute to cold adaptation rather than merely representing a generalized stress response [32]. *Pyramimonas gelidicola*, an Antarctic chlorophyte, provides another example. Two antifreeze protein isoforms, Pg-1-AFP and Pg-2-AFP, with predicted molecular masses of 26.4 and 27.1 kDa, respectively, exhibited thermal hysteresis of 0.60 ± 0.02 °C and 0.25 ± 0.02 °C at approximately 15 mg mL^−1^. These findings demonstrate that cold adaptation in polar microalgae can involve structurally diverse ice-binding proteins with distinct activities rather than a universal antifreeze mechanism [34]. Studies of cold-responsive gene expression further indicate that adaptation involves coordinated regulation of cellular metabolism. Li et al. [35], for example, reported differential regulation of cold-inducible genes, including *hiC6*, *hiC12*, *rpl10a* and *hsp70*, together with reduced expression of chlorophyll a/b-binding protein genes in *Chlorella vulgaris* exposed to 4 °C. Because *C. vulgaris* is a conventional biotechnology species rather than an established psychrophilic extremophile, this observation should be interpreted as evidence of a cold-stress response rather than proof of psychrophily. This distinction illustrates the importance of separating natural ecological adaptation from experimentally induced physiological acclimation [35].

### 3.3. Acidophilic and Alkaliphilic Microalgae and Cyanobacteria

Acidophilic and alkaliphilic photosynthetic microorganisms are adapted to environments characterized by extreme proton concentrations. Acidophilic taxa occur in environments such as acid mine drainage and geothermal systems, whereas alkaliphilic and alkalitolerant taxa are associated with soda lakes and other high-pH environments. For example, *Galdieria sulphuraria* is a well-characterized thermoacidophilic red microalga capable of growth under highly acidic and elevated-temperature conditions [29]. Cyanidiales are particularly informative because their adaptation reflects simultaneous selection by low pH, high temperature and, in some habitats, elevated concentrations of dissolved metals. Members of this lineage can inhabit hot acidic springs at approximately pH 0–4 and temperatures approaching 56 °C, making them representative polyextremophilic eukaryotes [27]. Acidophilic microalgae maintain intracellular homeostasis through proton extrusion, membrane-associated transport systems, metabolic reorganization and mechanisms that limit the intracellular accumulation of toxic ions. In *Cyanidium caldarium*, intracellular pH is maintained near neutrality despite the steep external proton gradient, with proton extrusion mediated by plasma-membrane ATPase activity [36]. Comparative genomic and transcriptomic analyses of *Chlamydomonas eustigma* further showed elevated expression of heat-shock proteins and plasma-membrane H^+^-ATPase, loss of metabolic pathways that could acidify the cytosol, and acquisition and multiplication of metal-detoxification genes. These findings illustrate that acidophily involves coordinated genetic and physiological restructuring rather than a single proton-pumping mechanism [37]. By contrast, alkaliphilic microorganisms must maintain intracellular proton gradients while acquiring inorganic carbon under conditions in which dissolved CO_2_ availability is restricted. Consequently, efficient bicarbonate utilization, carbon-concentrating mechanisms, ion transport and regulation of intracellular pH are central components of adaptation to alkaline environments. The capacity to maintain photosynthetic carbon assimilation under high pH may therefore provide an ecological advantage, but it should not automatically be interpreted as evidence of improved carbon-capture performance at process scale. The diversity of pH responses is illustrated by *Limnomonas gaiensis*, which can tolerate exposure across approximately pH 3–11, although optimal survival occurs between pH 5 and 8 and responses are strain-specific. Because the strains investigated originated from freshwater lakes rather than strongly acidic or alkaline habitats, they are more appropriately described as broadly pH-tolerant or extremotolerant rather than obligate acidophiles or alkaliphiles, as shown in Table 1.

### 3.4. Halophilic and Halotolerant Microalgae

Halophilic microalgae occur in hypersaline environments such as salt lakes, solar salterns, hypersaline ponds, lagoons and concentrated brines. Their adaptation is particularly well illustrated by species that originate from naturally hypersaline habitats and maintain growth under high salt concentrations. Examples include *Aphanothece halophytica*, *Dunaliella* spp. and several halophilic green algae, diatoms, and red algae. Villanova et al. [38], for example, isolated *Dactylococcopsis salina* from a saltern pond in Trapani, Sicily, whereas Abomohra et al. [39] isolated *Tetraselmis elliptica* from the hypersaline Bardawil Lagoon in North Sinai, Egypt.

Adaptation to hypersalinity involves the coordinated regulation of water potential, intracellular ions, and compatible solutes [40]. *Dunaliella salina* is a particularly well-characterized example because it can withstand a broad range of NaCl concentrations and rapidly regulate intracellular glycerol in response to changes in external osmolarity [41,42]. Transcriptomic analyses further demonstrate rapid and extensive changes in gene expression following hyperosmotic stress, including regulation of glycerol metabolism, transporters, carbohydrate metabolism and other stress-responsive pathways. This response should nevertheless be interpreted carefully because glycerol accumulation is a mechanism of osmotic adjustment, not necessarily evidence of increased overall productivity. Similarly, an increase in the cellular concentration of a metabolite does not necessarily translate into increased volumetric productivity because growth may simultaneously decline. This distinction is important when assessing the biotechnological value of halophilic organisms in later sections [43]. Halophilic microorganisms can also regulate intracellular Na^+^ and K^+^ through selective transport systems, ion pumps and associated membrane processes. These mechanisms contribute to maintenance of osmotic and electrochemical homeostasis and may operate together with compatible-solute accumulation rather than as independent responses. Li et al. [44] reported changes involving H^+^-ATPase, potassium transport and Na^+^/K^+^-ATPase-associated processes in *Nannochloris* under saline–alkaline stress. Because the experiment involved imposed saline–alkaline conditions, however, the findings are more appropriately interpreted as evidence of stress acclimation unless the strain’s natural habitat independently establishes extremophily [44].

**Table 1:** Physiological and molecular responses of microalgae to acidic and alkaline conditions.

Microalgae	Experimental/Environmental Condition	Physiological/Molecular Changes	Evidence Classification	Reference
*Desmodesmus* sp., *Heterochlorella* sp.	Culture conditions producing pH 3.5–6.7 in the presence of heavy metals	Exposure to pH 3.5 increased protein and lipid contents	Stress-response experiment; not sufficient alone to establish acidophily	[45]
*Phaeodactylum tricornutum*	pH 5.5–10.5	Acidic conditions altered growth and stimulated pathways associated with pH regulation/ion transport, fatty-acid metabolism, photosynthesis and carbohydrate metabolism	Stress-response experiment	[46]
*Nannochloropsis oceanica*	Short- and long-term acidification through elevated pCO_2_	Short-term acidification increased soluble carbohydrate, protein and unsaturated fatty acids; prolonged acidification altered β-oxidation, Calvin-cycle and lipid pathways	Stress-response experiment	[47]
*Coccomyxa onubensis*	Acidic, Fe-rich conditions	Increased carotenoids, xanthophylls and phenolic compounds under Fe-associated oxidative stress	Naturally acidotolerant species; experimental response	[48]
*Limnomonas gaiensis*	pH 3–11	Broad pH tolerance; optimal survival at pH 5–8; strain-specific responses and altered starch/oil accumulation	Extremotolerant/pH-tolerant organism	[49]
*Chlamydomonas eustigma*	Naturally acidic environments; experimental pH gradient	Elevated H^+^-ATPase and heat-shock proteins; loss of pathways that acidify the cytosol; metal-detoxification genes	Naturally acidophilic species; genomic evidence	[37]
Acidophilic and alkaliphilic microalgae	Acidic and alkaline cultivation conditions	Differences in growth and CO_2_ uptake across pH conditions	Comparative physiological experiment	[50]
*Pseudochlorella* sp.	Isolation from sulfuric acid mine drainage; pH 1–7 cultivation	Adaptation to pH 3–5 associated with increased intracellular lipid droplets	Naturally acid-associated organism	[51]
*Galdieria phlegrea*	Urban wastewater cultivation	Increased lipid content under the tested conditions	Application study; not evidence of extremophily alone	[52]

### 3.5. Radiation-Responsive Microalgae

Radiation-responsive microalgae can withstand or respond to ionizing or UV radiation and the associated oxidative stress. Examples include *Chlamydomonas reinhardtii*, *Dunaliella salina*, *Haematococcus pluvialis*, *Chlorella* spp., and cyanobacteria such as *Arthrospira platensis*, *Nostoc* spp. and *Scenedesmus obliquus*. These organisms can activate DNA-repair, antioxidant and metabolic responses following radiation exposure, but such responses do not necessarily indicate that they evolved to thrive under chronically irradiated environmental conditions. For example, Dubey et al. [53] reported that ionizing radiation enhanced the antimicrobial activity of *C. reinhardtii* extracts through radiation-induced transgene expression and molecular modification of bioactive compounds [53]. This study demonstrates the potential of radiation as an experimental elicitor and metabolic modifier rather than evidence that *C. reinhardtii* has evolved sophisticated defenses specifically for survival under high-radiation environments. The adaptation and stress-response mechanisms associated with radiation exposure include DNA-damage repair, antioxidant defense, protection of photosynthetic machinery and metabolic reprogramming. Low-dose ionizing radiation can also induce stress-priming or hormetic-like responses in some microalgae. For example, low-dose irradiation of *C. reinhardtii* has been reported to stimulate growth and increase chlorophyll, protein, starch and lipid contents, accompanied by changes in DNA-damage-response and metabolic pathways [54]. Table 2 therefore summarizes the physiological and molecular antioxidant, DNA-repair and protective mechanisms reported in microalgae following radiation exposure, while distinguishing experimentally induced radiation responses from evidence of natural radiation adaptation.

### 3.6. Integrated Molecular Basis of Extremophilic Adaptation

Although the adaptive mechanisms described above differ among environmental extremes, they converge on several fundamental cellular requirements including maintenance of macromolecular stability, control of intracellular ion and proton gradients, preservation of membrane function, protection against oxidative damage, repair of macromolecular damage and redistribution of metabolic flux [15]. Extremophilic adaptation is therefore better understood as a systems-level phenotype than as a collection of independent stress-response pathways. Multi-omics approaches are increasingly useful for resolving this complexity because comparative genomics can identify lineage-specific genes and gene families associated with adaptation; transcriptomics reveals condition-dependent regulatory programs; proteomics establishes whether transcriptional changes are translated into functional proteins; and metabolomics captures downstream changes in carbon allocation, redox balance and compatible-solute production. Integrating these layers can help distinguish constitutive traits associated with an extremophilic lifestyle from transient responses induced by laboratory stress, particularly when coupled with appropriate ecological, physiological and comparative controls. Recent advances in algal and cyanobacterial omics further demonstrate the value of linking molecular variation to ecological function across extreme habitats [55]. This distinction is particularly relevant for biotechnology because the presence of an adaptive trait such as glycerol accumulation, proton extrusion, antioxidant production, antifreeze activity or radiation-induced metabolic remodeling does not by itself establish technological superiority. Its biotechnological significance depends on whether the trait produces a measurable advantage in growth, product productivity, process robustness, resource use or product quality under a defined operating regime. Thus, the adaptive phenotype should be considered a biological basis for technological potential rather than evidence of industrial feasibility [16].

**Table 2:** Physiological and molecular responses of microalgae to experimentally induced radiation.

Microalgae	Experimental Condition	Physiological/Molecular Changes	Evidence Classification	Reference
*Chlamydomonas reinhardtii*	X- and γ-irradiation	Dose-dependent changes in macromolecular composition and DNA-damage responses	Radiation-response experiment (RRE)	[54]
*Chlamydomonas reinhardtii*	0–400 Gy γ-irradiation	Differential regulation of genes associated with DNA repair, carbohydrate and energy metabolism and photosynthesis	RRE	[56]
*Chlamydomonas reinhardtii*	γ-irradiation	ROS formation, PSII protein modification and altered photosynthetic performance	RRE	[57]
*Dunaliella salina*	UV-B exposure	Dose-dependent effects on growth, nutrient uptake and ROS-associated responses	RRE	[58]
*Haematococcus pluvialis*	250–5000 Gy γ-irradiation	Altered astaxanthin and lipid production following irradiation	Experimental mutagenesis/elicitation (EME)	[59]
*Haematococcus pluvialis*	1000–5000 Gy γ-irradiation	Altered biomass and astaxanthin production under subsequent stress conditions	EME	[60]
*Chlorella vulgaris*	X-ray irradiation	Radiation-dependent changes in lipid and pigment profiles	RRE	[61]
*Haematococcus pluvialis*	UV-B exposure	Increased astaxanthin and oxidative-stress responses	RRE	[62]
*Chlorella sorokiniana*	1–20 Gy X-irradiation	Low doses induced hormetic metabolic activation and lipid-associated transcriptional responses	Experimental radiation priming	[63]
*Tisochrysis lutea*	γ-irradiation	Dose-dependent growth responses and hormetic effects	RRE	[64]

## 4. Environmental Functions and Circular Resource Utilization of Extremophilic Microalgae and Cyanobacteria

### 4.1. Ecosystem Services Role and Significance of Extremophilic Microalgae

The unique physiological and biochemical adaptations of extremophilic microalgae and cyanobacteria confer a survival advantage under environmental conditions that constrain many other photosynthetic microorganisms, allowing them to contribute to primary production, nutrient cycling, contaminant attenuation, and habitat functioning in extreme ecosystems. These functions need to be distinguished from the technological potential of extremophiles because the occurrence of a stress-tolerance trait does not necessarily translate into a measurable ecosystem service or a superior treatment process. Extremophilic microalgae and cyanobacteria can contribute to ecosystem functioning in hypersaline, acidic, alkaline, thermal, polar, and metal-rich environments, where their persistence enables photosynthetic carbon fixation and biomass production under conditions that exclude many conventional phototrophs [15,65]. Their ecological significance therefore lies primarily in maintaining biological activity and biogeochemical fluxes under environmental constraints, rather than in an inherent capacity to provide greater ecosystem services than non-extremophilic microalgae. The persistence of photosynthetic activity under extreme conditions can support primary production and provide organic carbon to associated microbial communities and higher trophic levels. Saini and Mona (2024), for example, isolated *Fischerella thermalis* from a hot spring in India and reported physiological responses to nitrogen availability involving pigment synthesis and cellular metabolism [66]. Similarly, Boutarfa et al. [67] reported substantial proportions of monounsaturated, saturated, and polyunsaturated fatty acids in *Coelastrella thermophila* var. *globulina* isolated from Algerian hot springs [67]. Such observations demonstrate physiological acclimation and biochemical flexibility, but should not be interpreted as direct evidence that these organisms enhance ecosystem productivity or biodiversity at larger spatial scales. Rather, they indicate mechanisms through which extremophilic phototrophs can persist and contribute to biogeochemical processes in habitats were environmental stress limits other primary producers. Extremophilic microalgae also have potential to contribute to contaminant attenuation, particularly in acidic, saline, or wastewaters where conventional biological treatment organisms may be less competitive. However, it is important to distinguish between bioremediation and contaminant removal. This is because the environmental fate of the contaminant depends on the underlying mechanism. Microalgal systems can remove contaminants through biosorption, bioaccumulation, biotransformation or biodegradation, as well as through physicochemical processes such as precipitation, volatilization, photolysis, or interactions with extracellular polymeric substances (EPS). Biosorption refers primarily to the passive binding of contaminants to cell surfaces or extracellular polymers; bioaccumulation involves metabolically mediated uptake into living cells; biotransformation denotes enzymatic conversion of a parent compound into one or more transformation products; and complete biodegradation or mineralization requires stronger evidence that the contaminant has been converted into simpler end products such as CO_2_, H_2_O, inorganic ions, or other demonstrably less hazardous products. These distinctions are particularly important when evaluating ecosystem benefits because transferring a contaminant from water to algal biomass does not constitute destruction and may create a secondary management requirement for the harvested biomass [68,69]. Evidence from naturally adapted extremophilic strains supports their capacity to tolerate and remove contaminants under conditions that are themselves extreme. Li et al. [70] for example, reported that *Nannochloropsis oceanica* removed 88.60%, 97.02%, 94.22%, and 98.02% of total phosphorus, total nitrogen, ammonia nitrogen, and chemical oxygen demand, respectively, from tofu wastewater under fed-batch cultivation [70]. Although this demonstrates substantial treatment performance, *N. oceanica* is not itself an extremophile solely because it was cultivated in wastewater; therefore, this result should be interpreted as evidence for microalgal wastewater treatment rather than as proof of an extremophilic advantage. By contrast, a strain of *Chlamydomonas acidophila* isolated from highly acidic, metal-rich mine-drainage ponds exhibited broad pH tolerance, high tolerance to Cd, Cu and Zn, and intracellular metal accumulation, with metabolic changes associated with Cu tolerance [71]. This example directly links natural adaptation to an extreme habitat with a potential remediation function. Transcriptomic analyses of *C. acidophila* further identified differential regulation of photosynthesis-, carbohydrate-metabolism-, and stress-response-related transcripts under elevated Cu, providing molecular evidence for the physiological basis of metal tolerance [72].

The thermo-acidophilic red microalga *Galdieria sulphuraria* provides another example in which extremophilic physiology intersects with wastewater treatment. Henkanatte-Gedera et al. [73] demonstrated that *G. sulphuraria* could grow in primary-settled urban wastewater and remove dissolved organic carbon, ammoniacal nitrogen, and phosphate, with removal efficiencies of 46–72%, 63–89%, and 71–95%, respectively, in a 700 L photobioreactor [73]. Importantly, this study provides evidence extending beyond flask-scale experiments to a 700 L field photobioreactor, but it remains a demonstration-scale application rather than evidence of ecosystem-scale restoration. More recent work has further shown that *G. sulphuraria* can tolerate acidic media containing Cd, Pb, Ni and Zn and remove these metals from aqueous systems, although removal efficiencies varied among metals and decreased in some mixed-metal conditions [74,75]. These findings indicate that extremophilic microalgae may be particularly useful for treatment streams in which low pH or elevated metal concentrations constrain conventional biological treatment, but comparative studies against non-extremophilic strains under identical operating conditions are required to establish whether extremophilia provides a consistent technological advantage. The mechanisms underlying heavy-metal attenuation involve interactions between the metal ions and the extracellular and intracellular components of the algal cell. Surface functional groups in cell walls and EPS can passively bind metal ions, whereas metabolically active cells may subsequently internalize and sequester metals through intracellular ligands, including thiol-containing compounds and phytochelatins. These processes should not be assumed to represent the same mechanism or to result in permanent metal removal from the environment. Kharel et al. [74] reported maximum removal efficiencies of 45.90%, 25.15%, 6.56%, and 28.44% for Cd(II), Pb(II), Ni(II), and Zn(II), respectively, using *G. sulphuraria* under acidic conditions [74]. Similarly, heterologous expression of phytochelatin synthase from *C. acidophila* in *Escherichia coli* increased tolerance to selected heavy metals, supporting a mechanistic role for phytochelatin-mediated metal binding. Nevertheless, evidence from heterologous systems should be distinguished from direct demonstrations of phytochelatin-mediated removal in intact extremophilic microalgal cultures [76]. For wastewater containing complex mixtures of nutrients, metals and organic contaminants, extremophilic microalgae may function as part of an integrated algal–bacterial treatment system rather than as isolated remediation agents. Nitrogen and phosphorus can be assimilated into biomass, while organic compounds may be transformed through algal metabolism, extracellular enzymes, associated bacteria, or indirect photochemical processes. Microalgal wastewater treatment therefore involves multiple interacting pathways, including nutrient assimilation, biosorption, bioaccumulation, biodegradation, biotransformation, photolysis and volatilization, and the relative contribution of each pathway depends on contaminant chemistry and operating conditions. Algal–bacterial consortia are particularly relevant for complex wastewater because bacterial partners can contribute complementary degradation pathways, while microalgal photosynthesis supplies oxygen and assimilates nutrients; however, the operational mechanisms and large-scale applicability of such systems remain incompletely resolved [68,69]. The attenuation of organic pollutants requires particular caution in interpreting ecosystem benefits. Extremophilic microalgae may contribute to the transformation of pharmaceuticals, pesticides and other organic contaminants through enzymatic and photochemical processes. Gérin and Remacle (2024), for example, reported an increase in laccase activity in *Chlamydomonas pitschmannii*, demonstrating the capacity of this organism to oxidize phenolic and non-phenolic substrates under appropriate conditions [77]. However, a decrease in the concentration of a parent pollutant should not automatically be interpreted as biodegradation, mineralization, detoxification or ecological restoration. Transformation products may persist or retain biological activity, and claims of harmless byproducts require identification of transformation products together with evidence of reduced toxicity or, preferably, demonstrated mineralization. Accordingly, the ecosystem-service contribution of extremophilic microalgae is best framed as the capacity to retain biological activity, participate in nutrient and contaminant fluxes, and facilitate pollutant attenuation under environmental conditions that are unfavorable to many conventional biological systems, rather than as an intrinsic guarantee of complete remediation. Overall, the available evidence supports three interconnected ecosystem functions of extremophilic microalgae and cyanobacteria: maintenance of primary production under environmental extremes, participation in nutrient and elemental cycling, and attenuation or transformation of selected contaminants. The strength of evidence, however, varies substantially among these functions. Ecological persistence and physiological adaptation are well supported for numerous extremophilic taxa, whereas direct evidence that their presence produces measurable ecosystem-level improvements remains comparatively limited. Likewise, most remediation studies remain laboratory- or engineered-system demonstrations, with relatively few studies establishing long-term performance under field conditions. Future assessments should therefore distinguish natural ecosystem functioning from engineered treatment performance and compare extremophilic strains with conventional microalgae under equivalent conditions. Such comparisons, together with mass balances, transformation-product analysis, toxicity testing and long-term field validation, are necessary to determine when extremophilic traits provide a genuine ecological or technological advantage rather than simply enabling survival under otherwise inhibitory conditions [78].

### 4.2. Extremophilic Microalgae and Cyanobacteria as Platforms for Circular Resource Recovery

The circular-bioeconomy potential of extremophilic microalgae and cyanobacteria lies in their capacity to couple stress-adapted biomass production with the recovery of multiple biological resources. Extremophilic microalgae and cyanobacteria offer potential advantages because their resilience may enable cultivation under saline, acidic, alkaline, or thermally challenging conditions that can constrain conventional production systems. However, stress tolerance alone does not establish lower costs or greater sustainability; any process advantage depends on biomass productivity, process stability, downstream processing requirements, and the value of the recovered products. Their cultivation in non-conventional water resources may additionally reduce competition for freshwater, although this benefit must be evaluated against the energy and material requirements of cultivation and biomass processing. Rather than focusing on remediation functions discussed above, the circular-bioeconomy perspective emphasizes the conversion of the resulting biomass and extracellular materials into multiple value-added products [23,79]. A major opportunity is the cascading fractionation of extremophilic biomass to recover proteins, lipids, carbohydrates, pigments, EPS, and other bioactive metabolites. High-value compounds can potentially be recovered first, followed by conversion of residual biomass into lower-value products such as biofuels, biochemicals, fertilizers, or other biomaterials. Such cascading approaches could improve resource-use efficiency by distributing economic value across several product streams rather than relying on a single end product. For example, stress-adapted organisms can accumulate compatible solutes, carotenoids, lipids, and other protective metabolites that provide targets for bioprospecting and downstream valorization. Nevertheless, increased metabolite content should be distinguished from volumetric productivity and commercial feasibility, because stress conditions can enhance product accumulation while simultaneously reducing biomass growth [80,81]. Extracellular polymeric substances provide an additional route for converting extremophilic biomass into functional materials. Ciempie et al. [82] reported greater Pb^2+^ binding by EPS from the extremophilic microalga *Vischeria magna* than by EPS from *Chlorella vulgaris* and *Parachlorella kessleri* [82]. Such findings indicate potential for developing algal EPS as biosorbents, bioflocculants, or other functional biomaterials; however, their technological relevance requires evaluation of production yield, chemical consistency, adsorption capacity, selectivity, regeneration, stability, and production cost. Thus, a demonstrated biochemical property should be considered evidence of functional potential rather than proof of scalable application [82].

Residual biomass can also be integrated into a biorefinery cascade through fermentation, anaerobic digestion, thermochemical conversion, or other routes to generate fuels, chemicals, or soil-related products. This approach can reduce residual waste and improve overall biomass utilization, but the environmental and economic benefits depend on the compatibility of successive fractionation steps and the energy required for harvesting, dewatering, drying, extraction, and purification. In particular, intensive downstream processing can offset the benefits associated with stress-resilient cultivation, highlighting the need to evaluate the entire production chain rather than individual biological advantages [83]. Biomass quality is another critical determinant of circular valorization. When extremophilic organisms are cultivated in contaminated waste streams, accumulated metals or other pollutants may restrict the use of the biomass for food, feed, nutraceutical, or pharmaceutical applications. Therefore, biomass intended for valorization requires contaminant characterization and appropriate safety assessment before high-value applications are considered. Where contamination limits direct use, the biomass may instead be directed towards non-food materials or energy recovery pathways with appropriate contaminant management [84]. The integration of extremophilic microalgae and cyanobacteria into circular-bioeconomy systems should therefore be evaluated as a linked sequence of biomass generation, selective product recovery, residual biomass utilization, and waste minimization rather than as an inherently sustainable process. Life-cycle assessment (LCA) and techno-economic analysis (TEA) are needed to determine whether the advantages of stress tolerance, use of non-conventional resources, and biochemical diversity translate into lower environmental burdens and viable production costs. Overall, current evidence supports extremophilic microorganisms as promising platforms for diversified biomass valorization, but the strongest evidence remains at laboratory and early process-development stages. Future research should prioritize integrated biorefinery configurations, long-term process stability, cascading extraction, biomass safety, comparative benchmarking, and LCA–TEA to establish whether their distinctive stress-adaptation traits provide a measurable advantage over conventional microalgal production systems [85,86,87].

### 4.3. Contribution of Extremophilic Microalgae and Cyanobacteria to Carbon Capture, Utilization, and Sequestration

Extremophilic microalgae and cyanobacteria have potential for carbon capture and utilization because their tolerance to high CO_2_ concentrations and other flue-gas stressors may facilitate biomass production under conditions that inhibit conventional strains. However, extremophily alone does not establish higher carbon-fixation efficiency, lower production costs, or permanent carbon sequestration. Their potential application in industrial settings, such as power plants and other flue-gas sources, therefore depends on strain-specific tolerance, biomass productivity, gas-transfer efficiency, contaminant tolerance, and process integration. For example, Cho et al. [88] reported stable growth in a dual-strain system containing *Galdieria sulphuraria* exposed to simulated flue gas containing NO, CO_2_ and SO_2_, achieving a combined CO_2_ biofixation rate of 793 mg L^−1^ d^−1^ [88]. This result demonstrates tolerance and CO_2_ biofixation under a defined experimental flue-gas condition, but does not by itself establish net greenhouse-gas mitigation or industrial-scale feasibility. Importantly, carbon capture, carbon fixation, carbon utilization, and carbon sequestration represent distinct processes: photosynthetic fixation transfers CO_2_ into biomass, utilization converts that biomass into products, whereas sequestration requires sufficiently long-term retention of carbon to prevent its rapid return to the atmosphere [89]. Accordingly, the majority of reported extremophilic microalgal systems should be described as CO_2_ capture/fixation or utilization platforms rather than permanent sequestration technologies.

The ability of some extremophilic microorganisms to maintain photosynthetic activity under elevated CO_2_, temperature, salinity, or pH may facilitate carbon fixation under conditions that constrain conventional production systems. Carbon-concentrating mechanisms (CCMs), including inorganic-carbon transport and carbonic anhydrase activity, contribute to intracellular CO_2_ availability and can support RuBisCO carboxylation under carbon-limited conditions. Nevertheless, the presence of CCMs is not sufficient evidence of superior whole-system CO_2_ removal, which is also governed by light availability, mass transfer, pH, temperature, nutrient supply, biomass concentration, and reactor configuration. Premaratne et al. [90] reported a CO_2_ fixation rate of 0.21 ± 0.02 g L^−1^ d^−1^ when *Desmodesmus* sp. was exposed to flue gas containing 15.50% CO_2_ under nitrogen limitation, accompanied by lipid and carbohydrate accumulation of 419.57 ± 31.52 and 327.65 ± 23.39 mg L^−1^, respectively [90]. These findings illustrate the potential for coupling CO_2_ fixation with biomass valorization, but the increase in product accumulation should not be interpreted as evidence of increased carbon-removal efficiency because nutrient limitation can alter both biomass productivity and cellular composition. Shokravi et al. [91] reported higher photosystem II and I efficiency when *Fischerella* sp. was exposed to an irradiance of 2 μmol photons m^−2^ s^−1^ under limited CO_2_ availability at pH 9 [91]. Similarly, Curien et al. [92] showed that organic-carbon supplementation altered intracellular carbon metabolism in *Galdieria sulphuraria*, demonstrating the metabolic flexibility of this organism rather than establishing a direct increase in atmospheric CO_2_ sequestration [92].

**Figure 1:**
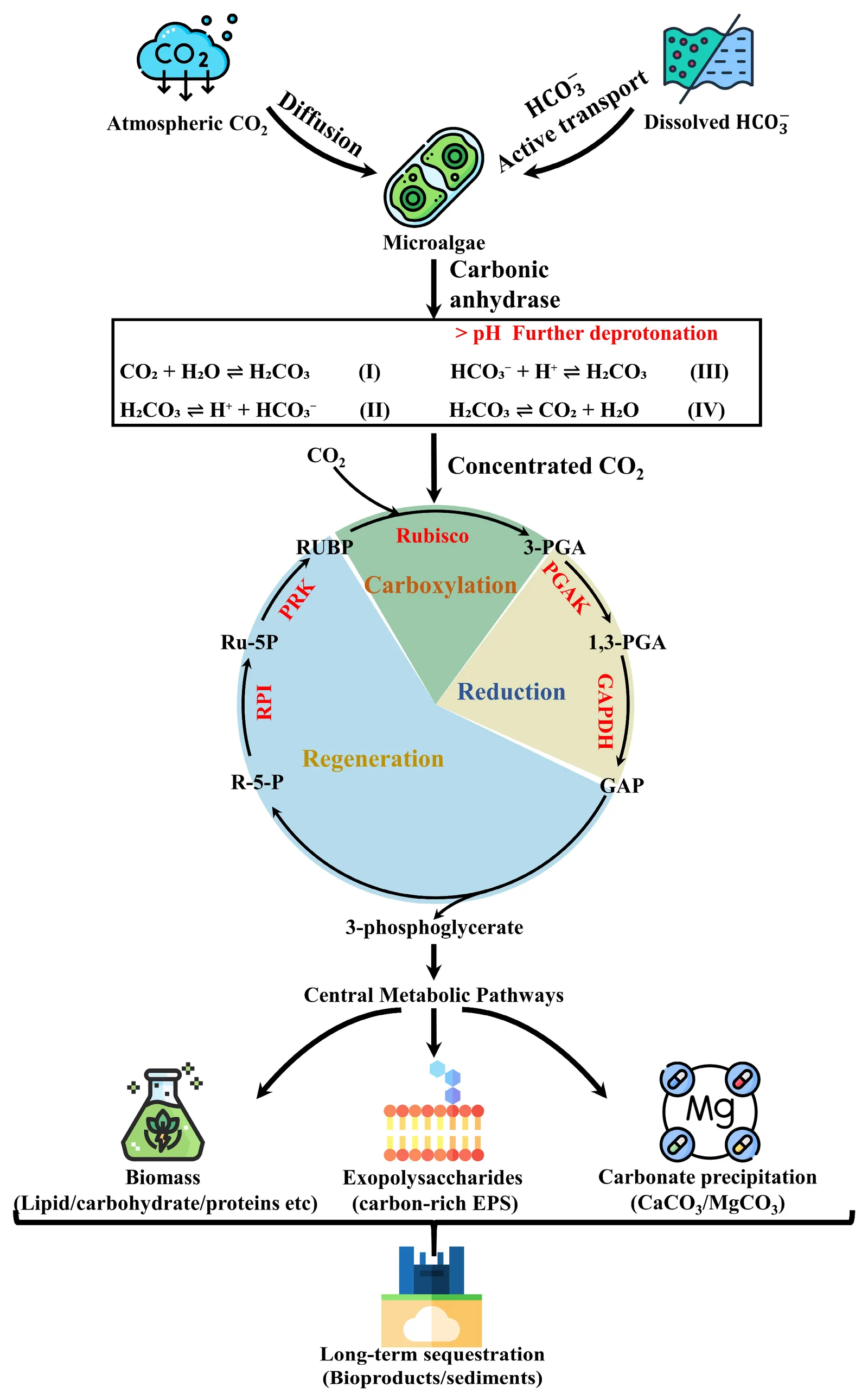
Role of carbon concentration mechanisms in carbon sequestration by extremophilic microalgae.

The main biochemical components of CCMs include carbonic anhydrases (CAs), which catalyze the reversible interconversion of CO_2_ and bicarbonate (HCO_3_^−^), thereby facilitating inorganic-carbon acquisition and delivery to sites of CO_2_ fixation (Figure 1) [93]. Their contribution to carbon fixation is nevertheless context-dependent because CA activity must be integrated with inorganic-carbon transport, cellular demand, light availability, and pH-dependent CO_2_ speciation. Other adaptations include the ability of some extremophilic microalgae to utilize multiple carbon sources. Occhipinti et al. [94] reported that *Galdieria sulphuraria* can grow mixotrophically and heterotrophically using lactose, glucose, or galactose as carbon sources [94]. This metabolic flexibility may improve carbon utilization and biomass formation when organic substrates are available, but heterotrophic or mixotrophic growth should be distinguished from net photosynthetic CO_2_ removal because externally supplied organic carbon can contribute substantially to biomass formation. Another proposed adaptation involves strain-specific changes in RuBisCO kinetics under extreme conditions. However, claims of enhanced CO_2_ affinity or reduced photorespiration should be restricted to systems for which these properties have been experimentally demonstrated. Steensma et al. [95] reported that RuBisCO from *Cyanidioschyzon merolae* exhibits kinetic properties compatible with carbon fixation under the organism’s thermoacidophilic conditions, highlighting the value of extremophilic enzymes for understanding carbon fixation under environmental stress [95]. From a climate-mitigation perspective, the fate of the fixed carbon is therefore critical. Biomass converted into fuels, rapidly degradable products, or short-lived chemicals can return much of the assimilated carbon to the atmosphere, whereas durable carbon products, mineralization, or appropriately managed long-term storage may provide more persistent carbon retention. Consequently, claims of “carbon sequestration” should be supported by carbon mass balances and evidence of storage permanence rather than inferred solely from CO_2_ fixation rates. [83,84] Moreover, the environmental performance of extremophilic CO_2_-fixation systems must be assessed at the process level. Energy demand for CO_2_ compression or delivery, mixing, illumination, temperature control, harvesting, dewatering, drying, and downstream conversion can substantially influence the net climate benefit. LCA and TEA are therefore essential for determining whether biological CO_2_ fixation produces a net environmental and economic advantage over alternative carbon-management technologies. Thus, the strongest near-term opportunity may lie in integrated systems that couple tolerant strains with concentrated CO_2_ sources and biomass valorization, while claims of permanent sequestration, net-negative emissions, reduced energy demand, or superior cost-effectiveness require validation at pilot and larger scales [96,97].

## 5. Bioprospecting of High-Value Bioproducts from Extremophilic Microalgae and Cyanobacteria

### 5.1. Extremophilic Microalgae and Cyanobacteria as Sources of High-Value Bioproducts: Bioprospecting Potential

**Figure 2:**
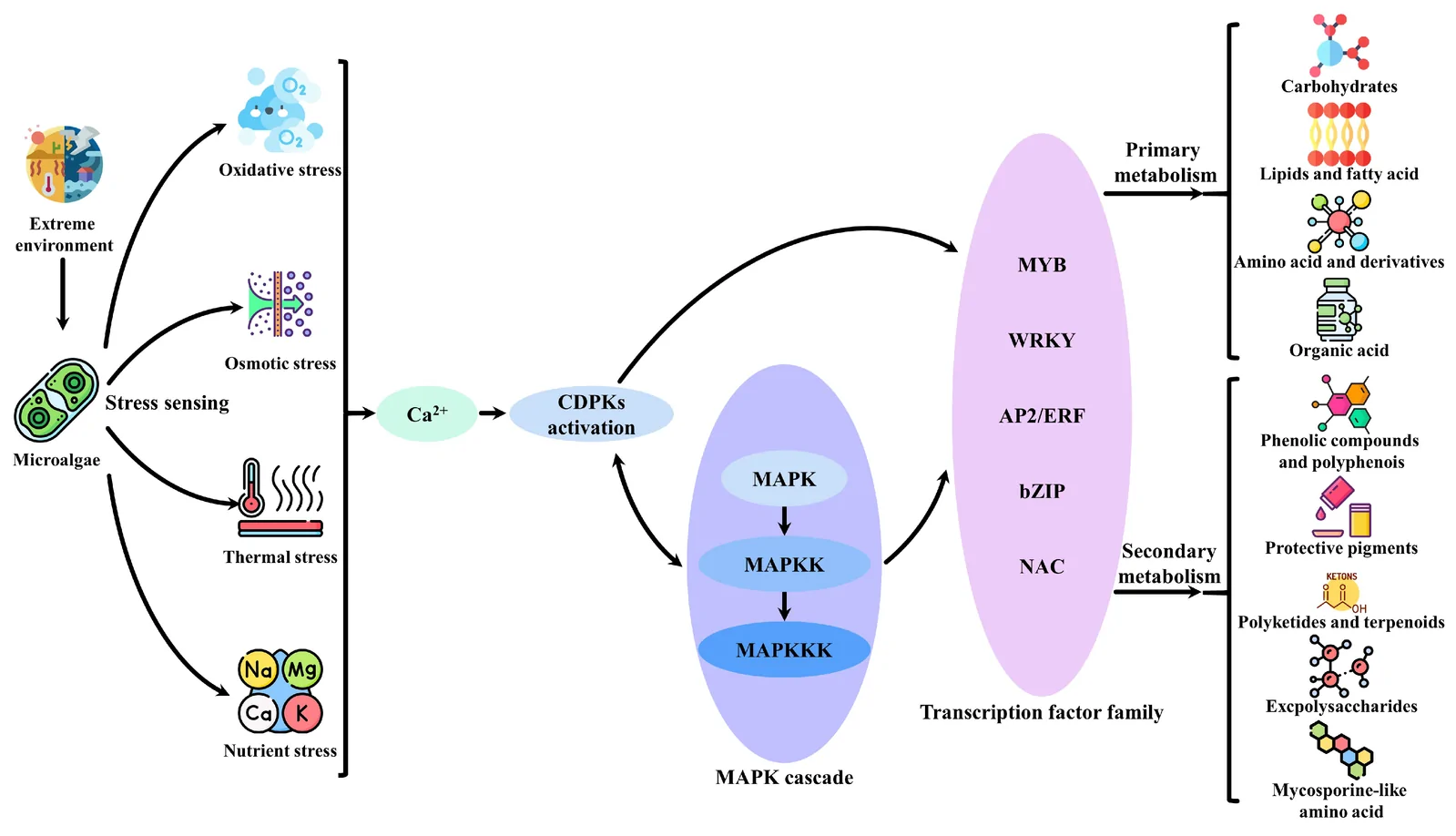
Mechanism of stress-triggered accumulation of metabolites in extremophilic microalgae.

The physiological and biochemical adaptations of extremophilic microalgae and cyanobacteria can redirect metabolic fluxes towards protective and storage compounds, creating a reservoir of chemically diverse molecules for bioprospecting. Their ability to thrive in extreme environments is associated with the production or accumulation of bioactive molecules including pigments, mycosporine-like amino acids (MAAs), phenolic compounds, terpenoids, lipids, carbohydrates, and extracellular polymeric substances (EPS) [23,98]. These properties provide a basis for identifying candidate molecules and enzymes with potential industrial value, but metabolite accumulation under laboratory stress should not be equated with demonstrated commercial production. The mechanisms underlying the formation of these metabolites are illustrated in Figure 2. Primary metabolism supplies carbohydrates, proteins, lipids and fatty acids, amino acids and derivatives, and organic acids, whereas stress-responsive and specialized metabolism can generate or modify pigments, MAAs, phenolic compounds, polyketides, and terpenoids [99].

### 5.2. Bioprospecting Potential of Lipids and Fatty Acids Derived from Extremophilic Microalgae

Extremophilic microalgae produce a wide range of lipid compounds whose composition can vary substantially with temperature, salinity, pH, carbon availability, and nutrient status. These changes can support membrane homeostasis and energy storage under environmental stress and may also generate commercially relevant fatty-acid profiles. Vítová et al. [100] reported that changes in pH altered lipid composition in *Galdieria sulphuraria*, including replacement of cellular phospholipids by betaine lipids [100]. Ahmed et al. [101] reported 22.28% neutral lipids and total lipids in *Dunaliella salina* exposed to 2 M NaCl [101]. Soru et al. [102] reported production of caprylic acid (C8:0) by *Coccomyxa melkonianii* under nitrogen starvation [102]. However, these stress-induced responses should be interpreted cautiously because nitrogen starvation is a metabolic induction strategy widely used in conventional microalgae and does not, by itself, establish extremophily. Similarly, Breuer et al. [103] reported TAG accumulation above 35% of dry weight in *Chlorella zofingiensis*, *Scenedesmus obliquus*, *Neochloris oleoabundans*, and *Chlorella vulgaris* under nitrogen deficiency [103]. These species are therefore better regarded as conventional microalgae subjected to nutrient stress rather than as evidence of extremophilic lipid production. For naturally extremophilic organisms, stress-induced lipid remodeling may nevertheless provide useful bioprospecting targets. Sakurai et al. [104] showed that autotrophic, mixotrophic, and heterotrophic cultivation of *Galdieria sulphuraria* altered the amounts and composition of glycogen and lipids [104]. Such metabolic flexibility may facilitate the production of tailored lipid fractions, although the optimal cultivation strategy must balance product concentration against biomass formation. Extremophilic microalgae also modify their fatty-acid profiles in response to environmental conditions, helping maintain membrane fluidity under thermal or osmotic stress [105]. López et al. [106] reported that carbon source and temperature affected PUFA accumulation in *Galdieria* sp., particularly arachidonic acid (C20:4) and eicosapentaenoic acid (C20:5) [106]. Kania et al. [107] further showed that *Coccomyxa subellipsoidea* acclimatized to a broad temperature range through reversible changes in unsaturated fatty-acid composition [107]. These findings demonstrate adaptive lipid remodeling and identify fatty-acid profiles of potential value for nutraceutical, chemical, or biomaterial applications, rather than establishing industrial-scale production. The biosynthesis of fatty acids in extremophilic microalgae follows the conserved biochemical principles observed across photosynthetic eukaryotes, while the enzymes, regulatory networks, and flux distributions can be adapted to environmental conditions. Fatty-acid synthesis involves acetyl-CoA carboxylase (ACCase), fatty-acid synthase (FAS), desaturases, elongases, and associated lipid-transfer pathways. Lu et al. [108] reported upregulation of ACCase expression in *Arthrospira* sp. exposed to 171.2 mM HCO_3_^−^ and 15 vol% CO_2_ [108]. Because *Arthrospira* is a cyanobacterium, it is considered separately from eukaryotic microalgae in this context. Bermejo et al. [109] reported that autotrophic cultivation of *Coccomyxa onubensis* at pH 2.5 resulted in 89.70 mg g^−1^ dry weight of fatty acid methyl esters, containing 28.70% palmitic acid and 37.8% linoleic acid [109]. By contrast, Jiao et al. [110] reported increased fatty-acid content and Δ5-desaturase-related gene expression in *Porphyridium purpureum* under nitrogen limitation [110]; because nitrogen limitation is not itself an extreme habitat condition, this result is best interpreted as stress-induced metabolic regulation rather than evidence of extremophilic adaptation. Gao et al. [111] reported that low temperature increased palmitoleic acid, eicosapentaenoic acid, and total fatty acids in *Xanthonema hormidioides* to maximum values of 23.64%, 2.49%, and 41.14% of dry weight, respectively [111]. Collectively, these studies demonstrate that environmental conditions can redirect fatty-acid metabolism, but comparisons should prioritize lipid productivity (mass of lipid produced per volume and time), rather than lipid content alone, because stress-induced increases in cellular lipid percentage may coincide with reduced growth.

The lipid profiles of extremophilic organisms may provide opportunities for bioenergy and oleochemical applications. However, biodiesel suitability should be evaluated using both lipid productivity and fuel-relevant fatty-acid composition, together with cultivation and downstream-processing requirements. Wan Afifudeen et al. [112] reported a cetane number of 61.9–64.4 and a degree of unsaturation of 45.3–57.4 for *Messastrum gracile* under nitrate-replete and nitrate-depleted conditions [112]. Although these values indicate potentially favorable fuel properties, *Messastrum gracile* is not itself evidence of an extremophilic advantage, and fuel quality does not establish economic feasibility. Tripathi et al. [113] reported a maximum lipid productivity of 22 ± 2.2 mg L^−1^ d^−1^ for *Coccomyxa* sp. under combined nitrogen, phosphorus, and sulfur limitation, with C16–C18 fatty acids predominating [113]. This distinction between lipid content and productivity is important because a commercially relevant strain must simultaneously achieve sufficient biomass productivity, lipid productivity, stability, and low-cost downstream recovery. Beyond biofuels, the value of extremophilic lipid bioprospecting may be greater for high-value fatty acids and specialized lipid molecules. PUFAs such as eicosapentaenoic acid and docosahexaenoic acid have established nutritional relevance. Asgharpour et al. [114] reported 13.08% total fatty acids in *Porphyridium cruentum* under defined light, temperature, and nitrate conditions [114]. Chalima et al. [115] reported DHA representing 35.6% of total lipids in heterotrophically cultivated *Crypthecodinium cohnii* using dark-fermentation effluent [115]. These studies illustrate the feasibility of using alternative cultivation conditions and waste-derived substrates to produce valuable fatty acids, but they should not be interpreted as evidence that extremophily itself confers superior PUFA productivity. Kuravi and Mohan [116] reported high proportions of myristoleic acid (C14:1) and heptadecanoic acid (C17:0) in mixotrophically cultivated *Messastrum gracile* [116]. Such compounds may have applications in food, feed, cosmetics, and specialty chemicals, but their practical value depends on purity, yield, safety, regulatory status, and the cost of extraction and purification. Extremophilic microalgae and cyanobacteria also contain diverse lipids with potential applications in specialty chemicals, surfactants, cosmetics, and biomaterials. Accordingly, the strongest bioprospecting rationale is not simply high lipid accumulation, but the discovery of unusual lipid structures, stress-responsive enzymes, and reproducible production phenotypes that cannot be readily obtained from conventional strains.

### 5.3. Bioprospecting Potential of Carbohydrate-Related Products Derived from Extremophilic Microalgae

The stress-adapted metabolism of extremophilic microalgae and cyanobacteria can support the production of structurally diverse extracellular and intracellular carbohydrates. Their metabolic flexibility allows some species to use different carbon sources under heterotrophic or mixotrophic conditions, while stress can redirect carbon towards storage or protective metabolites [117]. EPS is a carbohydrate of particular interest, particularly sulfated and fucosylated polymers, whose composition and structure can determine their physicochemical and biological properties. Mansour et al. [118] reported that sulfated EPS from *Halamphora* sp. contained 76.33 ± 1.80% carbohydrates, 5.44 ± 0.08% uronic acids, and 7.56 ± 0.86% sulfate [118]. Soanen et al. [119] reported EPS productivity of 0.096 g L^−1^ in *Porphyridium marinum* [119]. These compositional differences highlight the importance of structural characterization because monosaccharide composition, molecular weight, sulfation pattern, branching, and linkage chemistry can strongly influence EPS functionality. Sulfated EPS may contribute to protection against osmotic, thermal, and desiccation stress by forming hydrated extracellular matrices and modifying the local physicochemical environment. Drira et al. [120] reported a positive correlation between elicitor activity and sulfate-group content in EPS from *Porphyridium sordidum* [120]. Dulong et al. [121] reported that EPS from *Glossomastix* sp. contained 40–56% fucose and 20–31% rhamnose, together with minor monosaccharides including uronic acids [121]. These findings support the value of extremophilic microorganisms as sources of structurally diverse polysaccharides, but biological activity measured in vitro should be distinguished from validated pharmaceutical or industrial efficacy. Extremophilic microalgae also accumulate intracellular carbohydrates such as starch, glycogen, and compatible solutes that function in carbon storage and osmotic protection. Chokshi et al. [122] reported a 50% increase in intracellular carbohydrate in *Acutodesmus dimorphus* under 200 mM NaCl stress [122]. Because *Acutodesmus dimorphus* is not necessarily an obligate halophile, this observation is better interpreted as a salt-stress response rather than evidence of extremophilic adaptation. Buckeridge et al. [123] reported a 37% increase in intracellular carbohydrate in *Galdieria* sp. under ammonium limitation [123]. Again, nutrient limitation should be distinguished from the environmental conditions defining an extremophile. Keshari et al. [124] reported production of glucosylglycerol by *Leptolyngbya* sp. and sucrose by *Chroogloeocystis siderophila*, both isolated from extreme environments, under elevated salinity; glucosylglycerol reached 101 ± 12 mg L^−1^ OD_730_ in *Leptolyngbya* sp. [124]. Huang et al. [125] also reported increased glucosylglycerol in *Arthrospira maxima* to 149.00 mg L^−1^ under 300 mM NaCl stress [125]. These compatible solutes are particularly relevant to bioprospecting because their physiological role is directly linked to stress adaptation and may provide opportunities for specialty chemicals, osmoprotectants, and other high-value products.

Intracellular carbohydrates may also serve as feedstocks for bioenergy, although carbohydrate accumulation must be considered alongside biomass productivity and conversion efficiency. Microalgal carbohydrates can be hydrolysed and converted into fermentable sugars and subsequently into bioethanol, biobutanol, or biogas. Shih-Hsin Ho et al. [126] reported theoretical bioethanol yields of 79.9% and 92.3% following enzymatic hydrolysis of *Chlorella vulgaris* biomass using separate hydrolysis and fermentation and simultaneous saccharification and fermentation, respectively. Because *Chlorella vulgaris* is a conventional microalga, this study provides a conversion benchmark rather than evidence of an extremophilic advantage. For extremophilic strains, future assessments should therefore compare carbohydrate productivity, hydrolysis efficiency, inhibitor formation, conversion yield, and downstream energy requirements against conventional feedstocks [126].

Carbohydrate-derived products may also have high-value biomedical and nutraceutical potential. Guehaz et al. [127] reported 80.94 ± 0.01% α-d-glucosidase inhibition at 10 mg mL^−1^, with an IC_50_ of 4.31 ± 0.20 mg mL^−1^, for sulfated EPS from *Chlorella* sp. isolated from a hypersaline Algerian lake [127]. This result demonstrates in-vitro bioactivity but does not establish efficacy in vivo or suitability as a therapeutic or food ingredient; further toxicological, pharmacokinetic, structural, and formulation studies are required. Similarly, Mousavian et al. [128] reported anticoagulant activity in sulfated polysaccharides extracted from *Chlorella sorokiniana* and *Chlorella* sp. Together, these findings illustrate the bioprospecting value of extremophilic and stress-adapted microalgal polysaccharides, while also emphasizing the need to progress from biochemical screening to standardized structural characterization, mechanism-of-action studies, safety evaluation, and scalable production before industrial or pharmaceutical claims can be made [128].

## 6. Discussion, Limitations and Future Directions

Extremophilic microalgae represent promising biological platforms for climate-resilient biotechnology rather than established futuristic tools for climate-change mitigation. Their resilience, metabolic plasticity, and ability to grow under high salinity, extreme pH, elevated temperature, or nutrient stress can facilitate biomass and metabolite production in environments unsuitable for many conventional crops or microalgal strains [129]. However, these advantages are context-dependent and do not inherently translate into lower environmental impacts or higher productivity at industrial scale. Their potential to utilize saline water, wastewater, or non-arable environments could reduce competition for freshwater and agricultural land, while simultaneously enabling resource recovery and bioproduct generation. For example, a pilot-scale study by Zhu et al. [130] reported that cultivation of *Dunaliella salina* in seawater pretreated through removal of 97.6% Ca^2+^ and 60.3% Mg^2+^, followed by CO_2_ supplementation to increase total inorganic carbon to 0.254 mol L^−1^, resulted in the production of 14.3 g β-carotene and 300 g biomass [130]. Wolf et al. [131], under outdoor conditions mimicking the Mediterranean summer climate, reported growth of *Dunaliella salina* at salinities of up to 110 g L^−1^ and β-carotene production of 25 mg L^−1^ (3 mg gCDW^−1^) [131]. These studies demonstrate the feasibility of stress-resilient cultivation, but they should not be interpreted as evidence that extremophilic systems are universally more sustainable than conventional cultivation platforms. The cultivation of extremophilic microalgae may also reduce contamination pressure under appropriately selective conditions. Hirooka and Miyagishima [132], for example, reported algal yields of 2.73 and 2.49 g dry weight L^−1^ for *Galdieria sulphuraria* and *Pseudochlorella* sp., respectively, when cultivated in acidic hot-spring water supplemented with inorganic nitrogen [132]. Nevertheless, cultivation performance remains highly dependent on strain, substrate composition, environmental control and process configuration.

The continued reliance on fossil fuels is a major driver of climate change, and microalgal CO_2_ capture has therefore attracted considerable attention. Extremophilic microalgae may offer a niche advantage for CO_2_ utilization where elevated temperature, salinity, acidity, or contaminant concentrations restrict conventional algal cultivation. Zhu et al. [133] reported maximum carbon-capture rates of 1.00 and 0.81 g L^−1^ d^−1^ when *Limnospira platensis* was exposed to 0.5 M bicarbonate at pH 12.5 [133]. Similarly, Mohler et al. [134] reported biomass productivities of 0.1–0.2 g L^−1^ d^−1^ for *Scenedesmus acutus* cultivated in a cyclic-flow photobioreactor designed to capture and recycle CO_2_ from an industrial source [134]. However, CO_2_ fixation should be distinguished from permanent carbon sequestration because biological fixation represents temporary incorporation of carbon into biomass unless the carbon is subsequently stored in durable products or otherwise prevented from rapid re-emission. Moreover, the overall climate benefit depends on electricity demand, CO_2_ delivery, cultivation infrastructure, harvesting, downstream processing, and the fate of the biomass. Life-cycle studies have shown that cultivation, harvesting and drying can dominate environmental burdens, emphasizing that high biological productivity alone is insufficient to demonstrate climate mitigation. Mu et al. [135], using life-cycle assessment, reported that wastewater-based cultivation of *Chlorella* sp. for biofuel production could outperform freshwater cultivation environmentally, although the outcome depended on wastewater characteristics [135]. More recent LCA studies similarly indicate that wastewater integration can reduce environmental impacts, but energy-intensive aeration, harvesting, and drying remain important hotspots.

Extremophilic microalgae can also accumulate lipids, carbohydrates, pigments, and other metabolites with potential applications in bioenergy, nutraceuticals, pharmaceuticals, and biomaterials. However, product-specific productivity, recovery efficiency, purity, stability and market value must be considered alongside biomass yield when evaluating their commercial potential. The accumulation of polyhydroxyalkanoates (PHAs), for example, has been reported in *Arthrospira platensis*, with Nguyen et al. [136] observing PHA levels exceeding 39% of total biomass under combined bicarbonate and propionate supplementation [136]. Such findings demonstrate biochemical potential but do not by themselves establish commercial feasibility, particularly because substrate costs, downstream recovery and process energy requirements can strongly influence economic performance. Similarly, extremophilic microalgal biomass and extracts may contribute to agricultural applications as biofertilizers or bio-stimulants. Castro et al. [137] reported that digested *Scenedesmus* sp. biomass supplied 21% phosphorus, 9% nitrogen, and 12% organic matter to soil [137]. Further field-scale validation is nevertheless required to determine nutrient-release dynamics, agronomic efficacy, contaminant safety and consistency across soil types and climatic conditions.

A major limitation of extremophilic microalgal bioprospecting is the narrow physiological window within which individual strains achieve optimal growth and product formation. Although tolerance to extreme conditions can reduce contamination by non-adapted organisms, it can simultaneously increase requirements for specialized infrastructure, materials, and process control. For example, *Galdieria sulphuraria* is adapted to acidic environments, whereas *Arthrospira platensis* requires specific combinations of temperature, alkalinity, salinity and nutrient availability for stable productivity [138]. Therefore, the assumption that extremophilic cultivation is inherently low-maintenance should be avoided. Maintaining extreme pH, salinity, or temperature on a scale can increase energy and chemical requirements, while variable wastewater composition and seasonal conditions can alter growth and product yields. The commercial feasibility of extremophilic platforms should consequently be established using integrated techno-economic analysis (TEA), life-cycle assessment (LCA), mass and energy balances, and sensitivity analysis rather than biological productivity alone. Recent microalgal LCA–TEA studies demonstrate that cultivation configuration, electricity demand, biomass concentration and downstream processing can substantially influence both environmental performance and production cost [139].

Future research should therefore prioritize the following directions:

- Integrating genomics, transcriptomics, proteomics, metabolomics and physiological phenotyping to identify conserved and strain-specific determinants of extremophile adaptation. Such datasets could guide targeted metabolic engineering and adaptive laboratory evolution to improve product selectivity while minimizing growth penalties.
- Developing controlled mixed-culture and microbiome-based systems to determine how extremophilic microalgae interact with bacteria and other microorganisms under realistic cultivation conditions. This is particularly important for evaluating stability, contaminant resistance, nutrient exchange, and potential ecological risks associated with environmental deployment.
- Coupling automated sensing, process control, and machine-learning models with cultivation systems to dynamically regulate pH, salinity, temperature, light, nutrient supply, and CO_2_ delivery. Such systems should be evaluated against conventional control strategies using energy consumption, productivity and economic performance as explicit benchmarks.
- Establishing predictive strain-to-process frameworks that integrate genomic traits, physiological responses, environmental variables and process economics to identify suitable cultivation niches and product targets. These models should be coupled with TEA and LCA to distinguish biologically feasible strategies from those that are genuinely environmentally and economically viable.

## 7. Conclusions

Extremophilic microalgae represent promising platforms for climate-resilient bioprospecting and circular biotechnology because their physiological and metabolic adaptations enable survival and bioproduct formation under conditions that constrain conventional production systems. Their stress-responsive pathways support the production of lipids, pigments, carbohydrates, proteins, and other bioactive metabolites with potential applications in pharmaceuticals, cosmetics, agriculture, and bioenergy. Their ability to utilize saline water, wastewater, and other non-conventional resources also offers opportunities to couple biomass production with resource recovery and CO_2_ utilization. However, these advantages remain strongly strain- and process-dependent, and their contribution to climate mitigation, carbon sequestration, and industrial sustainability requires validation through integrated techno-economic, life-cycle, and scale-up assessments. Future research should therefore prioritize linking extremophile physiology and metabolic engineering with process optimization and environmental assessment to translate demonstrated stress tolerance into viable and resource-efficient bioprocesses.

## Data Availability

No new data were created or analyzed in this study. Data sharing is not applicable to this article.
